# Eosinophilic Cholangitis and Cholangiopathy: A Sheep in Wolves Clothing

**DOI:** 10.1155/2010/906496

**Published:** 2010-11-07

**Authors:** Catherine Nashed, Sujit Vijay Sakpal, Victoria Shusharina, Ronald Scott Chamberlain

**Affiliations:** ^1^School of Medicine, St. George's University, Grenada, West Indies; ^2^Department of Surgery, Saint Barnabas Medical Center, 94 Old Short Hills Road, Livingston, NJ 07039, USA; ^3^Department of Pathology, Saint Barnabas Medical Center, 94 Old Short Hills Road, Livingston, NJ 07039, USA; ^4^Department of Surgery, Saint Barnabas Medical Center, University of Medicine & Dentistry of New Jersey, 150 Bergen Street, Newark, NJ 07103, USA

## Abstract

*Background*. Eosinophilic cholangitis (EC) is a rare benign disorder of the biliary tract which can cause biliary obstruction. Similar to other disease processes involving the bile ducts, this disorder can pose a difficult diagnostic challenge as it can mimic cholangiocarcinoma. *Methods*. A systematic search of the scientific literature was carried out using PubMed to access all publications related to EC. Search keywords that were utilized included “eosinophilic cholangitis,” “etiology,” “treatment,” and “obstructive jaundice.” *Results*. Twenty-three cases of EC have been reported. Nineteen patients (82.6%) who presented with EC remain disease-free; 15 of these 19 patients (78.9%) with followup time remain disease-free at a mean of 9.7 months (range, 2–24 months). *Conclusion*. EC is a rare form of biliary obstruction which can masquerade as a malignancy. Unlike cholangiocarcinoma, EC occurs more commonly in younger patients and in men. Most patients will require surgical treatment.

## 1. Introduction

A variety of biliary conditions can mimic cholangiocarcinoma (CCA) and the precise pathologic distinction between benign and malignant causes of common hepatic duct (CHD), and proximal biliary tract obstruction remains a challenging clinical problem. In addition to malignant causes such as hilar CCA, lymphoma, gallbladder carcinoma, and metastases, benign causes of biliary obstruction include, but are not limited to: autoimmune pancreatitis-associated sclerosing cholangitis, extrahepatic primary sclerosing cholangitis (PSC), Mirizzi syndrome, inflammatory strictures secondary to choledocholithiasis, idiopathic benign focal stricture, and acquired immune deficiency syndrome cholangitis [1–6]. Approximately 10% of patients who undergo surgery for hepatic hilar strictures are found to have benign disease [1, 2]. Hadjis and colleagues initially coined the term “malignant masquerade” in 1985 to emphasize how benign entities can be difficult to distinguish from hilar CCA, in both clinical presentation and radiological appearance [1]. 

Eosinophilic cholangitis (EC) is an extremely rare benign disorder of the biliary tract which can result in biliary obstruction [3–5, 7, 8]. This disorder must be distinguished from CCA, which can be difficult since it is characterized by a dense transmural eosinophilic infiltration of the bile duct. A comprehensive literature review identified only 22 cases of EC [2–4, 8–24]. In this paper, we discuss an additional case involving a 33-year-old man who presented with obstructive jaundice and a mid-bile duct stricture (Bismuth-Corlette Type III). Extensive radiologic and endoscopic evaluation failed to yield a pathologic diagnosis, and common bile excision with Roux-en-Y hepaticojejunostomy, cholecystectomy, and portal lymphadenectomy was performed. Histopathology revealed a dense eosinophilic infiltration of the extrahepatic bile duct that was consistent with EC. A review of the literature and a discussion of the clinical presentation, diagnosis, management, and prognosis of patients with EC are provided.

## 2. Case Report

**Figure 1:**
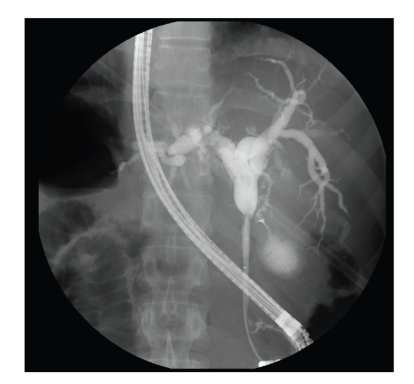
Endoscopic retrograde cholangiopancreatography (ERCP) showing focal stricture of the common hepatic duct at the level of entry in to the cystic duct.

A 33-year-old Caucasian man presented with a two-month history of fatigue, jaundice, severe pruritis, and steatorrhea. He noted a 10–15 pound weight loss over the prior two months but denied nausea, vomiting, abdominal pain, fever, or chills. Past medical history was significant for hyperlipidemia, with no history of biliary tract disease or ulcerative colitis (UC). Family history was negative for inflammatory bowel disease (IBD) or gastrointestinal (GI) malignancies. There was no history of foreign travel or significant allergic or atopic reactions. Physical exam disclosed no abnormalities except for scleral icterus and mild tenderness on deep palpation in the right upper quadrant (RUQ) of his abdomen. Pertinent laboratory tests revealed elevated liver function tests (LFTs): aspartate aminotransferase, 75 IU/L; alanine aminotransferase, 208 IU/L; alkaline phosphatase, 337 IU/L; gamma-glutamyl transferase, 1166 IU/L; total bilirubin, 5.2 mg/dl, and direct bilirubin, 4.65 mg/dl. Amylase and lipase levels were 52 IU/L and 250 IU/L, respectively. Total white blood cell count was 5.4 × 10^3^/mm^3^, with a differential cell count of 56% neutrophils, 32% lymphocytes, and 2% eosinophils. The patient's hemoglobin, hematocrit, and platelet counts were 13.5 gm/dl, 41.6%, and 354 × 10^3^/mm^3^, respectively. Hepatitis virus screening, tumor and immunological markers including carcinoembryonic antigen (CEA) and carbohydrate antigen 19-9 (CA19-9), and antinuclear (ANA), antimitochondrial (AMA), and antismooth muscle (ASM) antibodies were all unremarkable. Immunoglobulin G (IgG) quantitative levels were also negative, all but ruling out autoimmune sclerosing cholangitis as a possible diagnosis. Ultrasonography (US) of the RUQ revealed a normal gallbladder with no evidence of gallstones and markedly dilated intrahepatic and extrahepatic bile ducts down to the level of mid-common bile duct (CBD). These findings were confirmed by a computed tomography (CT) scan of the abdomen and pelvis and a magnetic resonance cholangiopancreatography (MRCP) which further demonstrated a focal stricture of the CHD at the level of the cystic duct entry (Figure 1). The left and right intrahepatic ducts measured 13 mm and 14 mm, respectively. The CBD and pancreatic duct were normal in caliber. An endoscopic retrograde cholangiopancreatography (ERCP) was performed, at which time the biliary stricture was dilated and a silastic biliary endoprostheis was inserted. Brush biopsy obtained during ERCP revealed no malignant cells. Esophagogastroduodenoscopy (EGD) revealed only mild reflux esophagitis. In order to exclude the possibility of concurrent undiagnosed UC, a colonoscopy with ileoscopy and biopsy was performed which revealed a normal colon with no evidence of colitis. An endoscopic US (EUS) demonstrated a mass in the proximal CHD with wall thickness of 4.7 mm. EUS-guided fine needle aspiration (FNA) of the mass showed marked inflammatory changes along with clusters of atypical ductal cells. The patient underwent spy cholangioscopy, which confirmed a CHD stricture, yet additional biopsy sampling for cytology and fluorescent *in situ *hybridization (FISH) yielded only atypical cells with no pathologic diagnosis. 

**Figure 2:**
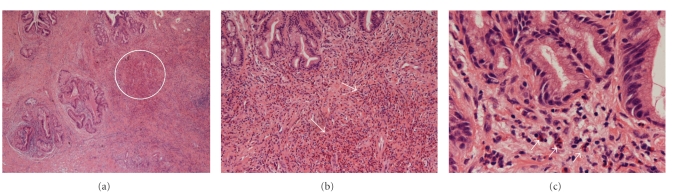
(a) Low power magnification displaying a common bile duct wall with bile duct mucosal glands, composed of columnar cells with basally oriented nuclei and subepithelial collagen. There is no epithelial atypia. The subepithelial area shows periductal fibrosis and pronounced diffuse inflammatory cellular infiltrate. White circle indicates infiltrate. (Hematoxylin and Eosin stain; (b) displaying 20x; (c) displaying 40x magnification). (b) and (c) Higher-power view demonstrating bile duct mucosal glands composed of columnar cells with basally oriented nuclei. In the subepithelial region, there is a dense infiltrate comprising predominantly of eosinophils, identified by their pathognomonic cytoplasmic granules and bilobed nuclei. The eosinophils are present diffusely within the stroma and around the glands. However, in some areas, these cells form clusters of four to five cells. The white arrows demonstrate the clusters of eosinophils. Along with the eosinophils, other inflammatory cells are also identified, including lymphocytes with scanty cytoplasm and small hyperchromatic dark blue nuclei as well as the larger neutrophils with their granular cytoplasm and multilobed nuclei. (Hematoxylin and Eosin stain; 1b displaying 20x; 1c displaying 40x magnification).

In order to exclude the possibility of a malignancy, the patient subsequently underwent an exploratory laparotomy, cholecystectomy, portal lymphadenectomy, and an *en bloc *resection of the entire CBD from the supraduodenal aspect to the hilar bifurcation. Frozen section revealed no evidence of malignancy. An intraoperative liver biopsy revealed normal hepatic architecture with no evidence of PSC or granulomas. The bilioenteric anastomosis was reconstructed via a Roux-en-Y hepaticojejunostomy. Histopathology revealed thickening of the CBD wall with periductal fibrosis and a pronounced inflammatory cellular infiltrate comprised almost entirely of eosinophils (Figures 2(a), 2(b), and 2(c)). The gallbladder was unremarkable and contained no gallstones. A total of 12 benign lymph nodes were harvested. The patient's postoperative recovery was uneventful, and he was discharged home on postoperative day five. At 40-month follow-up, the patient remains asymptomatic with normal LFTs.

## 3. Results

Twenty-three cases of EC have been documented, including the current case. Available demographic, clinic, and follow-up data are presented in Table 1. Among this group are 14 men and nine women (M : F = 1.6 : 1) with an overall mean age of 39.4 years (range, 13–67 years). The mean age among men was 35.4 years (range, 13–55 years), and the mean age among women was 45.8 years (range, 16–67 years). Abdominal pain was the most common presenting complaint (74%), followed by jaundice (61%). Among the 23 patients, 16 (69.6%) demonstrated peripheral eosinophilia while seven (30.4%) had normal serum eosinophil counts. Eight of the 23 patients (34.8%) had complete resolution of symptoms with surgery alone, seven patients (30.4%) improved with the use of oral corticosteroids, and another six patients (26.1%) achieved complete resolution with a combination of surgery and oral corticosteroids. One patient (4.3%) achieved symptom resolution with hydroxyurea treatment, after repeated high-dose steroid therapy was only temporarily successful. Lastly, one patient (4.3%) who presented with EC secondary to infection with *Echinococcus *(hydatid disease) achieved complete resolution of symptoms with albendazole treatment for a total of two weeks followed by surgical treatment which consisted of aspiration of the hydatid cyst and a left hepatic lobectomy. Fifteen of the 19 patients (78.9%) with reported follow-up time remain disease-free with a mean follow-up of 9.7 months (range, 2–24 months).

## 4. Discussion

EC was first reported by Leegaard in 1980 [9]. Although the cause of this disorder remains unknown, it appears to be a benign, self-limiting disease. Underlying infections with *Enterobacter aerogenes *in one patient and *Candida albicans *in another have been postulated as a cause for the eosinophilic infiltration [2, 10]. However, each of the two above-mentioned patients had undergone prior instrumentation of the biliary tree; [2, 10] therefore, it seems most likely that the biliary infections were secondary events rather than the primary cause of the inflammatory process. It has also been noted that gallstones within the biliary system may act as irritant foci which results in eosinophilic infiltration in some patients [10, 25]. Several studies have demonstrated a link between hypereosinophilia and bile duct fibrosis. Wong and colleagues suggested that eosinophils produce transforming growth factor-*β*, a cytokine capable of inducing fibrosis [26]. Although a number of theories have been postulated, the precise pathogenesis of the eosinophilic infiltration is poorly understood. 

Eosinophilic cholangiopathy is part of a larger spectrum of disorders characterized by eosinophilic infiltration of tissues and organ systems with or without concomitant peripheral eosinophilia. In this spectrum of disorders, all patients have one thing in common: unexplained eosinophilic proliferation. However, the severity and prognosis of the disorder vary between patients. The most severe pathology in this spectrum of diseases is idiopathic hypereosinophilic syndrome (IHES) which involves eosinophilic infiltration of the bone marrow and other organs. IHES is characterized by (1) persistent eosinophilia of 1.5 × 10^9^/L for at least six months or any eosinophilia leading to death within six months; (2) lack of a recognized cause for the eosinophilia, including parasitic infections, collagen vascular diseases, and allergies; (3) organ system involvement or dysfunction due to eosinophilic infiltration or eosinophilia-associated damage [27, 28]. Multiorgan involvement is described in the majority of patients who present with biliary tract involvement and may include infiltration of the pancreas, liver, GI tract, ureters, and kidneys [4, 12–14]. There is no clear relationship between IHES and EC, which is not usually described as part of this syndrome. It remains unclear as to the pathophysiology of eosinophilic recruitment to the biliary tract in IHES. However, it seems that the presence of these eosinophils is directly related to the disease process, possibly via direct cytotoxicity, as they may release free radicals or tissue-damaging proteins [11]. The diagnosis of end-organ involvement is essential in IHES as the course of the disease may be variable. The disease may rapidly progress, eventually causing hepatotoxicity and fibrosis, and ultimately leading to the need for liver transplantation [11]. If caught early, however, corticosteroid therapy has been beneficial in up to 69% of patients with IHES [11]. 

Eosinophilic gastroenteritis (EGE) is a separate disease in this spectrum characterized by eosinophilic infiltration of the wall of the GI tract. EGE is thought to be associated with EC and may result from an allergic mechanism in 37%–41% of cases [3, 29]. EGE most commonly affects the stomach and proximal small bowel but may involve nearly every GI organ [29, 30]. EGE is characterized by (1) eosinophilic infiltration of one or more segments of the GI tract; (2) the presence of GI symptoms; (3) no recognizable cause for the eosinophilic infiltration; (4) no extradigestive involvement; (5) variable peripheral eosinophilia [30]. Although the most common symptoms of EGE are nausea, vomiting, diarrhea, and abdominal pain, the clinical manifestations of EGE vary depending on the area of the GI tract involved. Mucosal involvement manifests as weight loss secondary to malabsorption, steatorrhea, iron-deficiency anemia, blood loss, and protein-losing enteropathy, whereas involvement of the muscularis propria presents with obstructive symptoms, and subserosal disease results in eosinophilic ascites [15, 30]. 

The diagnosis of EC is a difficult one and is based solely on histological findings. Matsumoto et al. have proposed the following criteria to correctly diagnose EC: (1) wall thickening or stenosis of the biliary system; (2) histopathological findings of eosinophilic infiltration; (3) reversibility of biliary abnormalities without treatment or following steroid treatment [3]. Although the presence of peripheral eosinophilia may be a clue to the diagnosis of EC, it is neither sensitive nor specific of dense eosinophilic infiltration of the bile duct [2]. Laboratory values, including tumor markers, are useful in distinguishing between benign and malignant biliary obstruction but are usually unable to determine the exact cause of a biliary stricture [6]. The accuracy of fast liver alkaline phosphatase isoenzyme in differentiating between benign and malignant causes of extrahepatic obstruction has been reported to be up to 80% [31]. Tumor markers, such as CEA and CA19-9, have been used to distinguish CCA from other causes of obstruction, but they exhibit highly variable sensitivity and specificity [31–33]. An elevated CA19-9 level is not specific for a malignant process, as high circulating levels are noted in a variety of benign conditions including ascending cholangitis, pancreatitis, and other benign cases of obstructive jaundice [31–33]. 

A variety of modalities are currently available to view and evaluate the biliary system. Noninvasive radiological modalities include US, contrast-enhanced CT (CECT) scan, magnetic resonance imaging (MRI), and MRCP can provide useful information about the level of obstruction, extent of biliary dilatation, and the presence of a mass or distant metastasis [34, 35]. A common, but nonspecific finding in the setting of EC is thickening of the bile duct wall on US or CECT, with or without biliary dilatation [4, 19]. MRCP may demonstrate an irregular narrowing of the bile duct, while invasive modalities such as ERCP or percutaneous transhepatic cholangiography (PTC) may reveal irregularities of the CBD wall and intrahepatic ducts [4, 19]. ERCP or PTC can also provide additional information as to the length and site of a biliary stricture and allows for tissue diagnosis via brush biopsy and cytology studies [35]. EUS is an alternative modality to ERCP and PTC which allows antegrade biliary access via an EUS-guided needle puncture into the biliary system. This would allow for safe cannulization of strictures, biliary drainage, and other treatments offered by ERCP in settings of surgically-altered biliary anatomy or biliary inflammation [36, 37]. Cytology is an important part of the diagnostic workup of patients with eosinophilic cholangiopathy in order to rule out the possibility of CCA. Although routine brush cytology during ERCP has a high specificity (75%), the sensitivity rates are highly variable, with ranges from 44% to 80% [38, 39]. The use of FISH examination is a valuable tool for the detection of malignancy in biliary tract strictures [39]. While traditional cytology analysis identifies abnormally-shaped cells, FISH utilizes fluorescently-labeled DNA probes to assess for chromosomal alterations or malignant cells. Although cytology appears to be more specific than FISH for the detection of malignant strictures (98% versus 91%, resp.), Kipp and colleagues demonstrated that the sensitivity of FISH was significantly greater than routine cytology for bile duct brushing samples [39]. 

Single-operator direct cholangioscopy SpyGlass system is a new addition to the arsenal of available technologies for visualizing the bile ducts. It provides direct visualization of the biliary tract and has been shown to improve the ability to distinguish malignant from benign strictures [39, 40]. In a study by Kurland et al., four of 17 patients with an initial diagnosis of benign biliary strictures on cytology were found to have malignant strictures with SpyGlass-directed biopsies [39]. Overall, spy-directed biopsies has a sensitivity and specificity of 62.5% and 100%, respectively, using positive cytology or surgical biopsy as the reference standard [39]. Despite these efforts, precise tissue diagnosis in the setting of EC is typically not possible, and surgery is usually necessary to exclude CCA. 

Although EC is a benign self-limiting disease, the difficulty involved with excluding malignancy, and the variable course of the disease, makes precise treatment recommendations difficult [4, 16, 25]. Two reported cases of EC experienced spontaneous regression of the CHD stricture on repeat cholangiography within three weeks without any specific treatment [10, 16]. Although the role of steroids and hydroxyurea remains unclear, there are several cases of successful treatment with oral corticosteroids alone [3, 4, 8] (refer to Table 1). The suggested mechanism of action for corticosteroids in the treatment of eosinophilic cholangiopathy is unknown, yet Butler et al. reported that a “diagnostic” trial of oral corticosteroids may be considered prior to surgical intervention [16]. Despite the success of nonsurgical therapy in some cases, surgery is an effective and a definitive means of treatment for EC [29] and is obligatory if malignancy cannot be excluded. Fourteen of the 23 published cases were treated surgically.

## 5. Conclusions

Obstructive jaundice due to a mid or proximal biliary stricture poses substantial diagnostic and management issues. EC is a benign process capable of masquerading as a malignancy and posing substantial diagnostic challenges. The diagnosis of EC is difficult to conclusively make and often requires an extensive workup as in the current case. Despite its rarity, EC should be considered when imaging modalities demonstrate a narrowing of the extrahepatic bile duct(s) with marked wall thickening, especially in the setting of peripheral eosinophilia. Based on evidence of disease regression with oral corticosteroids, a course of oral corticosteroids is the initial recommended therapy. If diagnostic uncertainty persists, however, surgery is mandatory and curative in all reported cases.

## Figures and Tables

**Table 1:** All reported cases of eosinophilic cholangitis.

Case	Study	Gender age (yrs)		Presenting symptoms	Eos.	Treatment	Status
		Age	Sex				
1	Rodgers et al., [2] 2001	50	F	Abdominal pain	No	Roux-en-Y, CE	NED, 2 mos
2	Matsumoto et al., [3] 2007	38	F	Abdominal pain, jaundice	Yes	CS	NED, 5 mos
3	Vauthey et al., [4] 2003	44	M	Abdominal pain, jaundice	Yes	CS	NED, 18 mos
4	Duseja et al., [8] 2005	16	F	Abdominal pain, jaundice	Yes	CS	NED, 12 mos
5	Leegaard, [9] 1980	46	M	Abdominal pain, jaundice	No	CE, CS	NED, 18 mos
6	Rosengart et al., [10] 1990	48	M	Abdominal pain, jaundice	No	CE	NED, 5 mos
7	Al-Abdulla et al., [11] 2000	42	F	Abdominal pain, jaundice	Yes	CE, CS	NED
8	Platt et al., [12] 1990	56	F	Jaundice, ureteric obstruction	No	CE	N/A
9	Schoonbroodt et al., [13] 1995	20	M	Jaundice, fever	Yes	CE, CS	Recurred in stomach
10	Grauer et al., [14] 1993	41	M	Abdominal pain, jaundice, fever	Yes	CS, Ursodiol	Recurred in kidney
11	Jimenez-Saenz et al., [15] 2003	67	F	Abdominal pain, jaundice	Yes	CE, CS	NED, 12 mos
12	Butler et al., [16] 1985	32	M	Abdominal pain	Yes	CE	NED, 24 mos
13	Tenner et al., [17] 1997	38	F	Abdominal pain	Yes	CE, CS	NED, 3 mos
14	Shanti et al., [18] 2001	33	M	Abdominal pain, jaundice	No	CE, hepatico-jejunostomy	NED, 3 mos
15	Shanti et al., [18] 2001	57	F	Abdominal pain, jaundice	No	Roux-en-Y	NED, 6 mos
16	Song et al., [19] 1997	48	F	Abdominal pain	Yes	CE, T-tube	NED, 9 mos
17	Scheurlen et al., [20] 1992	28	M	Abdominal pain, diarrhea	Yes	Hydroxurea	NED
18	Chen et al., [21] 2009	55	M	Jaundice	Yes	Roux-en-Y, CE, CS	NED, 9 mos
19	Jeyamani et al., [22] 2007	13	M	Fever	Yes	CS	Recurred in liver
20	Jeyamani et al., [22] 2007	26	M	Fever, pruritis	Yes	CS	NED, 6 mos
21	Sussman et al., [23] 2008	52	M	Abdominal pain, pruritis	Yes	CS, AZT, UDCA	NED
22	Raptou et al., [24] 2009	24	M	Abdominal pain, fever	Yes	Aspiration, Albendazole	NED
23	Current study, 2009	33	M	Jaundice, pruritis	No	Roux-en-Y, CE, portal lymph-adenectomy, common bile excision	NED, 13 mos

Note. Eos: Eosinophilia; CS: Corticosteroids; CE: Cholecystectomy; AZT: Azathioprine; UDCA: Ursodeoxycholic acid; N/A: Not available; NED: No evidence of disease.
